# Post-traumatic cognitions and quality of life in terrorism victims: the role of well-being in indirect versus direct exposure

**DOI:** 10.1186/s12955-018-0923-x

**Published:** 2018-05-15

**Authors:** Miriam Bajo, Amalio Blanco, Maria Stavraki, Beatriz Gandarillas, Ana Cancela, Blanca Requero, Darío Díaz

**Affiliations:** 1 Department of Medical Psychology, Universidad de Castilla la Mancha, Ciudad Real Medical School, Camino de Moledores S/N, 13071 Ciudad Real, Spain; 2 Department of Social Psychology, Universidad Autónoma de Madrid, Calle Ivan Pavlov, 6, 28049 Madrid, Spain; 3 Department of Psychology, Universidad Francisco de Vitoria, Carretera Pozuelo-Majadahonda, Km 1.800, 28223 Pozuelo de Alarcón, Spain; 4 Department of Psychology, Centro Universitario Villanueva, Calle Costa Brava, 2, 28034 Madrid, Spain

**Keywords:** Well-being, Quality of life, Post-traumatic cognitions, Terrorism, Indirect-direct exposure

## Abstract

**Background:**

The effect of indirect (versus direct) exposure to a traumatic event on the quality of life of terrorist attack victims has received considerable attention in the literature. However, more research is required to examine whether the symptoms and underlying processes caused by both types of exposure are equivalent. Our main hypothesis is that well-being plays a different role depending on indirect vs. direct trauma exposure.

**Methods:**

In this cross-sectional study, eighty direct victims of 11-M terrorist attacks (people who were traveling in trains where bombs were placed) and two-hundred indirect victims (individuals highly exposed to the 11-M terrorist attacks through communications media) voluntarily participated without compensation. To test our hypothesis regarding the mediating role of indirect exposure, we conducted a biased corrected bootstrapping procedure. To test our hypothesis regarding the moderating role of direct exposure, data were subjected to a hierarchical regression analysis.

**Results:**

As predicted, for indirect trauma exposure, well-being mediated the relationship between post-traumatic dysfunctional cognitions and trauma symptoms. However, for direct trauma exposure, well-being moderated the relationship between post-traumatic dysfunctional cognitions and trauma symptoms.

**Conclusions:**

The results of our study indicate that the different role of well-being found between indirect (causal factor) and direct exposure (protective factor) should be taken into consideration in interventions designed to improve victims’ health.

## Background

The effect of exposure to a traumatic event on the health of terrorist attack victims has received considerable attention in the literature [1]. One of the most analyzed aspects is direct vs. indirect exposure to trauma. For example, in a study conducted after 11-S terrorist attacks (four coordinated terrorist attacks by al-Qaeda on the United States on September 11, 2001), the Post-traumatic Stress Disorder (PTSD) rate in a sample of 109 workers was 6.4%, for those directly exposed to a disaster site (World Trade Center) and 4.6% for those indirectly exposed, as revealed through survivor narratives [2]. Likewise, other studies about 11-S attacks have found a relationship between indirect exposure to trauma and the development of PTSD [3, 4], especially in vulnerable populations such as children [5]. In the same way, indirect exposure to terrorist attacks via the media (i.e. conventional or social media) increased stress and trauma of general population (e.g. 11-S terrorist attacks [6]; 2015 terrorist attacks in Paris [7]). In fact, previous research indicates that indirect exposure to a traumatic event appears to elicit similar symptoms and response patterns as direct exposure (i.e., re-experiencing, strategic avoidance, emotional numbing and hyperarousal symptoms [8]). It should be noted, however, that the prevalence of PTSD is higher among people who are directly exposed to the event [1].

Indeed, these findings have been taken into consideration in the revised definition of PTSD in the DSM-5, which explicitly includes indirect exposure to trauma (e.g. through a family member or close friend) as a possibility to meet criterion A. However, indirect exposure through media is still not included. Indirect exposure has been implicitly accepted since the DSM-IV through the use of “confronted with” language (i.e. the person experienced, witnessed, or was confronted with an event or events that involved (DSM IV A1 criterion [9]) (for a review of the evolution of the DSM definition of the stressor criterion see [10]). Although indirect exposure to trauma is now explicitly accepted as a possibility to meet PTSD criteria, more research is required to examine whether the symptoms and underlying processes caused by direct vs. indirect (and through the media) exposure to trauma are equivalent.

With this in mind, many theories about the underlying processes involved in the development of PTSD have focused on the analysis of cognitive elements, postulating that traumatic events produce changes in the victim’s thoughts and beliefs (e.g. Cognitive Model of PTSD [11]; Emotional Processing Theory [12]; Shattered Assumptions Theory [13]). In this sense, a new disorder has been proposed in the ICD-11: Complex Post Traumatic Stress Disorder (CPTSD). The symptom profile of CPTSD includes the core PTSD symptoms plus additional symptoms related with these thoughts and beliefs (e.g. negative self-concept [14]). Although cognitive theories differ when specifying the relevant beliefs related to the traumatic experience, they can be classified into three core groups: beliefs about the self, beliefs about the world, and trauma-related beliefs (e.g. its sequelae). For example, Epstein [15] and Janoff-Bulmann [16] proposed theories about how the traumatic events could break core beliefs (i.e. the world is benign, the world is meaningful, the self is worthy, or people are trustworthy), and thus increase mental health problems (because these beliefs are necessary for a positive psychological functioning). However, only a few trauma victims develop psychopathology (e.g. PTSD; [17, 18]) and many who initially develop PTSD recover over time without clinical treatment [19]. To explain these results, Foa and her colleagues suggested that PTSD is a consequence of disruptions in the normal processes of recovery related to dysfunctional cognitions (Emotional Processing Theory, EPT [12, 20]). To measure these trauma-related beliefs about self and world, Foa et al. [21], developed the Post-traumatic Cognitions Inventory (PTCI).

Research indicates that the changes in victim’s thoughts and beliefs caused by terrorists are strongly related to Psychological (PWB [22]) and Social Well-Being (SoWB [23]). For example, having *negative cognitions about the self* (a dimension of the PTCI) is a criterion that indicates a lack of *self-acceptance* (a dimension of PWB that implies having positive thoughts toward oneself [24]). *Social acceptance* (a dimension of SoWB) requires positive cognitions about the world [23], just the opposite of having *negative cognitions about the world* (other dimension of PTCI). In fact, several studies have found that exposure to trauma reduces victims well-being [25]. In accordance with this strong relationship, the objective of the present research was to analyze the role of well-being on the effect of post-traumatic cognitions on victims’ mental health (i.e. PTSD symptoms). Our first hypothesis (H1) is that a possible explanation for the higher prevalence of PTSD among individuals who were directly (vs. indirectly) exposed to a terrorist attack is that this kind of exposure produces strong dysfunctional cognitions about the self and about the world, and a higher reduction of victims’ well-being, which in turn increases the risk of developing PTSD. Moreover, our second hypothesis (H2) is that both direct and indirect exposure to a terrorist attack affect victims’ well-being differently. Our expectation is that indirect exposure to a terrorist attack causes negative cognitions about the self and the world, thus creating a sense of internal or external threat [26]. However, perceptions of threat may lack sufficient strength to generate strong dysfunctional cognitions. In this context, our expectation (H2a) is that indirect exposure will affect especially well-being (versus post-traumatic cognitions), and therefore well-being (i.e. PWB and SoWB) will mediate the relationship between negative cognitions and PTSD. In contrast, (H2b) our expectation is that direct exposure to a terrorist attack will generate strong dysfunctional cognitions that are an important risk factor in the development of PTSD. Therefore, we expect that well-being (i.e. PWB and SoWB) will moderate the relationship between negative cognition and PTSD, and thus emerge as a protective factor against the development of PTSD.

## Methods

### Participants

Two-hundred and eighty participants between 18 and 78 years old voluntarily participated in the study without compensation. Participants were 199 women (71%) and 81 men (29%) with a mean age of 28.75 years (*SD* = 13.57). The maximum educational level reached to 18.6% of primary education, 63.2% higher no university education, 16.4% hold a university degree and 1.8% a PhD. Eighty participants were *direct victims* of 11-M terrorist attacks. These attacks were nearly simultaneous, coordinated bombings against the train system of Madrid that resulted in 191 fatalities and more than 1800 injuries. These participants were recruited via letter of invitation explaining the project and the voluntary nature of participation. Participants were selected to meet the study criteria of 1) directly experiencing the traumatic event (DSM-V PTSD A1 Criteria) (all participants were traveling in one of the four trains in which bombs were placed); 2) no diagnosis of mental disorders (except PTSD) or another medical condition (except minor injuries that did not require hospitalization) at the time the study was conducted. The remaining two-hundred people were recruited via local newspapers advertisements from the general population of Madrid (Spain). The requirements to participate in the study were: 1) a high exposure to the 11-M terrorist attacks through traditional communication media; 2) not having any relative or friend directly affected by the attack; 3) no diagnosis of mental disorders or general medical condition (except PTSD) at the time the study was conducted. Perceived exposure of volunteers was measured using the following item: “How long have you been exposed to 11-M terrorist attack through traditional communication media (television, newspapers, radio programs…)?” Responses were made on 6-point scale (1 = “no time at all”, 6 = “all the time”). Participants who answered 5 or 6 fulfilled criterion 1 and were considered capable to participate in the study. Six-hundred eighty-seven applications that complied with these criteria were received, from which 200 were selected using a simple random sampling method. Although these participants were highly exposed to the 11-M terrorist attacks indirectly through communication media (*M* = 5.60; *SD* = .49), they did not fulfill the DSM 5 criterion for indirect exposure.

### Procedure

Participants completed the study three to six months after the attacks. This study was part of a research project funded by the Spanish Ministry of Education and Science, and was approved by the ethics committee of the coordinating university “Comité de Ética de la Investigación de la Universidad Autónoma de Madrid” (SEJ2006–14894). *Direct victims* completed the study within the psychological care protocol of 11-M Association of Victims. First, all participants completed an informed consent form, assuring them that all information they provided would remain confidential and anonymous. Following this, to reduce environmental influence [27], all participants were placed in individual lab cubicles and then provided with the experimental materials. Participants were provided with four questionnaires, which were presented in one of two orders to account for possible effects due to the order of presentation. Half the participants completed a booklet containing the Davidson Trauma Scale, followed by the Post-traumatic Cognitions Inventory. Then, in order of appearance, the Social Well-being Scales and the Psychological Well-being Scales. The other half completed the two groups of questionnaires in the reverse order.

### Measures

#### Trauma intensity

The Davidson Trauma Scale (DTS [28]; validated in Spanish by Bobes et al. [29]) linked with 11-M terrorist attacks, was used to obtain a general dimensional measure of trauma intensity. The DTS is a 17-item self-report questionnaire of post-traumatic stress symptoms, developed for use with trauma survivors. Each of the 17 items correspond to the 17 DSM-IV symptoms of PTSD and can be categorized as follows: items 1–4, 17 (criteria B, intrusive re-experiencing); items 5–11 (criteria C, avoidance and numbness); and items 12–16 (criteria D, hyperarousal). For each item, trauma survivors rate both frequency and severity using 5-point (0–4), Likert-type scales. In the current study, Cronbach’s *α* for the DTS-total score was .96 (*M* = 49.07; *SD* = 27.81), DTS-B = .89 (*M* = 16.19; *SD* = 9.07), DTS-C = .88 (*M* = 16.65; *SD* = 11.12) and DTS-D = .91 (*M* = 16.56; *SD* = 9.90).

#### Post-traumatic cognitions

The Post-traumatic Cognitions Inventory (PTCI [21]; validated in Spanish by Blanco, Díaz, Gaborit, & Amaris [30]), was used to measure post-traumatic cognitions. The PTCI is a 36-item self-report scale that yields three factors: negative cognitions about self (NCS), negative cognitions about the world (NCW), and self-blame (SB). According to the objectives, in the current study only the first two factors (NSC and NCW) were used. The PTCI possesses good internal consistency and factorial validity, and discriminant ability to differentiate people with and without PTSD [21]. Responses to the 28 items that comprised the two factors (NSC and NCW) were recorded on a 7-point scale ranging from 1 (strongly disagree) to 7 (strongly agree). In the present study, Cronbach’s *α* value for the NCS scale was .94 (*M* = 2.80; *SD* = 1.28) and NCW = .85 (*M* = 3.98; *SD* = 1.30).

#### Well-being

To measure PWB, participants responded to the Psychological Well-being Scales [22], validated in Spanish by Diaz and colleagues [31]. The instrument consists of six scales (autonomy, self-acceptance, positive relations, control of the environment, purpose in life and personal growth) and is reflected by one general factor. Participants responded to 39 items on a scale ranging from 1 (strongly disagree) to 6 (strongly agree). The proposed six-dimensional structure with a second order general factor has been tested using confirmatory factor analysis with Spanish samples [25, 31–33]. Based on the existence of one general factor, we computed only the sum of the 39 items as a global indicator of psychological well-being (Cronbach’s *α* = 92; *M* = 4.25; *SD* = .71). Also, participants completed Keyes’ Social Well-being Scales [23] validated and translated to Spanish [34]. This instrument consists of five scales (social integration, social acceptance, social contribution, social actualization and social coherence), which in previous studies have shown good internal consistency [23]. The proposed five-dimensional structure with a second order general factor has been tested using confirmatory factor analysis with Spanish samples [35]. In the present study we computed only the sum of all items as a global indicator of social well-being (Cronbach’s *α* = .90; *M* = 4.56; *SD* = .94). Participants responded to 25 items on a scale ranging from 1 (strongly disagree) to 7 (strongly agree).

### Data analysis

**Fig. 1:**
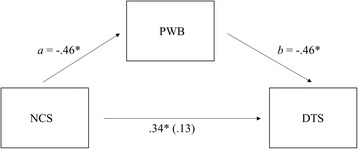
Psychological Well-being as a mediator between Negative Cognitions about Self and Post-traumatic Stress Symptoms (Indirect Exposure). Figure in the parenthesis (i.e., .13) is the direct effect of Thought Format X PCS on Well-being while accounting for the effect through the indirect path (* indicates *p* < .05)

**Fig. 2:**
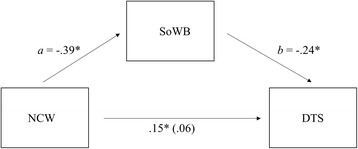
Social Well-being as a mediator between Negative Cognitions about World and Post-traumatic Stress Symptoms (Indirect Exposure). Figure in the parenthesis (i.e., .06) is the direct effect of Thought Format X PCS on Well-being while accounting for the effect through the indirect path (* indicates p < .05)

In order to analyze well-being and traumatic intensity differences between direct and indirect exposure to terrorist attacks (H1) we conducted different ANOVAS introducing age, sex and education as covariates. No significant gender, sex or education differences were found on any of the measures in the study. Thus, gender, sex and education are not discussed further. Pearson correlations were used to examine the relationships between all questionnaires. To test our hypothesis regarding the mediating role of indirect exposure (H2a), data were analyzed using two different approaches. First, we used the classical four steps method proposed by Baron and Kenny [36] and a Sobel Test, Aroian Test and Goodman Test. In order to provide a complementary test of mediation we conducted a biased corrected bootstrapping procedure with 10,000 bootstrap re-samples using Hayes PROCESS macro (model 4; see Figs. 1 and 2). PROCESS is a computational procedure for SPSS and SAS that implements moderation or mediation analysis as well as their combination in an integrated conditional process model [37–39]. Finally, to test our hypothesis regarding the moderating role of direct exposure (H2b), data were subjected to a hierarchical regression analysis. We introduced predictor variables at the first step, then added a computed interaction term at the second step.

## Results

### Direct exposure versus indirect exposure to terrorist attacks (H1)

Table 1 presents Pearson correlation coefficients among DTS, NCS (PTCI), NCW (PTCI), PWB and SoWB for direct and indirect exposure victims. Confirming expectations, post-traumatic cognitions and well-being were strongly related, particularly when we used the same individual versus social approach (PWB-NCW and SoWB-NCS).

**Table 1:** Pearson’s correlations and 95% confidence intervals of DTS, NCS (PTCI), NCW (PTCI), PWB and SoWB

	DTS	NCS	NCW	PWB	SoWB
Direct Exposure Victims					
DTS		.71** [.56 .82]	.63** [.45 .76]	−.41** [−.61–.16]	−.40** [−.59–.17]
NCS			.73** [.60 .82]	−.66** [−.79–.48]	−.43** [−.61–.21]
NCW				−.56** [−.72–.35]	−.42** [−.60–.20]
PWB					.63*[.44 .76]
SoWB					
Indirect Exposure Victims					
DTS		.34** [.21 .46]	.15* [.01 .28]	−.49** [−.59–.38]	−.25** [−.38–.12]
NCS			.49** [.38 .59]	−.46** [−.57–.34]	−.32** [−.44–.19]
NCW				−.20** [−.32–.05]	−.39** [−.50–.26]
PWB					.49** [−.59–.38]
SWB					

*p < .05

**p < .01

In line with prior research [1], there was an effect of exposure (direct versus indirect) on the DTS. The ANOVA showed that direct victims reported more traumatic intensity (*M* = 78.83, *SD* = 29.96) than did indirect victims (*M* = 39.74, *SD* = 19.46), *F* (1, 277) = 139.99, *η*_*p*_^2^ = .347, *p* < 0.001. According to our first hypothesis, direct exposure victims informed strong post-traumatic cognitions about the self (NSC) (*M* = 3.98, *SD* = 1.36) and the world (NCW) (*M* = 4.71, *SD* = 1.13) than indirect exposure victims (*M* = 2.37, *SD* = 1.23; *M* = 3.70, *SD* = 1.25), *F* (1, 277) = 122.56, *η*_*p*_^2^ = .311, *p* < 0.001; *F* (1, 277) = 37.34, *η*_*p*_^2^ = .120, *p* < 0.001. Finally, direct victims reported less psychological well-being (*M* = 3.61, *SD* = .75) and social well-being (*M* = 3.84, *SD* = 1.09) than indirect victims (*M* = 4.45, *SD* = .57; *M* = 4.83, *SD* = .72), *F* (1, 277) = 85.62, *η*_*p*_^2^ = .256, *p* < 0.001; *F* (1, 277) = 73.11, *η*_*p*_^2^ = .216, *p* < 0.001.

### Indirect exposure: the role of well-being in post-traumatic cognitions and PTSD symptoms relationship (H2a)

We propose that indirect exposure to trauma causes changes in victims’ thoughts and beliefs [16, 40–42]. In turn, this produces idiosyncratic negative appraisals that create a sense of internal or external threat [11] that affect well-being, although this threat may lack sufficient strength to generate strong dysfunctional cognitions. Thus, we predict that well-being mediates the relationship between post-traumatic dysfunctional cognitions and DTS. More specifically, we expect that PWB (an individual well-being construct [35] mediates the relationship between NCS and DTS, and that SoWB (a macrosocial well-being construct [23]) mediates the relationship between NCW and DTS.

According to the results of classic mediation analyses, NCS was associated with more DTS, *t* (198) = 5.15, *B* = .34, *p* < .01. Also, there was a significant effect of NCS on PWB *t* (188) = − 7.07, *B* = −.46, *p* < .01. Finally, in a simultaneous regression, the relationship between PWB and DTS was significant, *t* (187) = − 5.93, *B* = −.46, *p* < .01, and the direct effect of NCS on DTS was no longer significant, *t* (187) = 1.92, *B* = .13, *p* = .06. Classic theory tests for indirect effects confirm full mediation (Sobel Test: 4.54; Aroian Test: 4.52; Goodman Test: 4.57; all *p* < .01). In order to re-examine whether PWB mediated the effect of NCS on DTS, we conducted a biased corrected bootstrapping procedure with 10,000 bootstrap re-samples using Hayes PROCESS macro (model 4) [38, 39]. This approach includes procedures that compute a 95% confidence interval (CI) around the indirect effect and mediation is indicated if this CI does not include zero. NCS was the independent variable, DTS was the dependent variable, and PWB was the mediating variable (see Fig. 1). NCS, PWB, and DTS were mean-centered. As predicted, the data revealed that the 95% confidence interval of the indirect effect (i.e., the path through the mediator) did not include zero (Indirect Effect a x b = .21, CI95% = from .10 to .31), thus mediation by PWB is supported [39]. In order to examine whether SoWB mediated the effect of NCW on DTS, we followed the same procedure. First, NCW was associated with more DTS, *t* (198) = 2.15, *B* = .15, *p* = .03. Additionally, there was a significant effect of NCW on SoWB *t* (193) = − 5.83, *B* = −.39, *p* < .01. Finally, in a simultaneous regression, the relationship between SoWB and DTS was significant, *t* (192) = − 3.13, *B* = −.24, *p* < .01, while the direct effect of NCW on DTS was no longer significant, *t* (192) = .47, *B* = .06, *p* = .64. Classic theory tests for indirect effects confirm full mediation (Sobel Test: 2.75; Aroian Test: 2.72; Goodman Test: 2.79; all *p* < .01) (see Fig. 2). Also, the data revealed that the 95% confidence interval of the indirect effect did not include zero (Indirect Effect a x b = .09, CI95% = from .03 to .15), therefore indicating that mediation by SoWB is also supported.

### Direct exposure: the role of well-being in post-traumatic cognitions and PTSD symptoms relationship (H2b)

Based on *Emotional Processing Theory* [12, 20], we proposed that direct exposure to a high-intensity traumatic event causes strong dysfunctional cognitions that in turn constitute an important risk factor in the development of PTSD. In these situations, given the close relationship between well-being (i.e. PWB and SWB) and post-traumatic cognitions (i.e. NCS and NCW), our hypothesis (H2b) is that well-being moderates the relationship between post-traumatic cognitions and PTSD symptoms. Therefore, well-being should emerge as a “protective factor” for individuals that are directly exposed to trauma. Specifically, we expect PWB to moderate the relationship between NCS and DTS and SoWB to moderate the relationship between NCW and DTS. To test our hypothesis, DTS was subjected to a hierarchical regression analysis. We introduced NCS and PWB (centered score) as predictor variables at the first step, and added a computed interaction term at the second step.

**Fig. 3:**
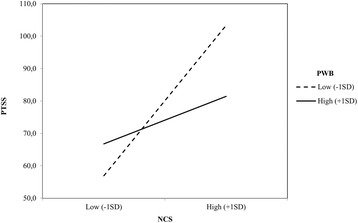
Psychological Well-being as a moderator of the effects of Negative Cognitions about Self on Post-traumatic Stress Symptoms (Direct Exposure)

The results of this analysis revealed that the main effect of NCS, *B* = .30, *t* (77) = 2.19, *p* = 0.03, was significant, but the main effect of PWB, *B* = −.17, *t* (77) = .76, *p* = .45, was not significant. Most relevant for purposes of the present research, the data revealed a significant NCS x PWB interaction, *B* = −.26, *t* (76) = − 2.09, *p* = .04. As depicted in Fig. 3, this interaction revealed that among participants low in PWB (− 1 SD), NCS were strongly related with DTS, *B* = .78, *t* (76) = 3.32, *p* = .01. This relationship was only marginally significant among those participants who reported high PWB (+ 1 SD), *B* = .25, *t* (76) = 1.71, *p* = .09.

**Fig. 4:**
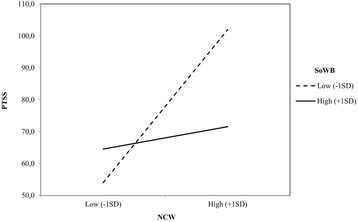
Social Well-being as a moderator of the effects of Negative Cognitions about World on Post-traumatic Stress Symptoms (Direct Exposure)

In order to examine whether SoWB moderated the effect of NCW on DTS, we followed the same procedure. The results of the hierarchical regression analysis revealed a significant main effect of NCW, *B* = .81, *t* (77) = 5.64, *p* = .01, and SoWB, *B* = −.38, *t* (77) = − 2.68, *p* = .01. As expected, the NCW x SoWB interaction was significant, *B* = −.36, *t* (76) = − 2.96, *p* = .01. This interaction showed that among participants low in SoWB (− 1 SD), NCW were related with DTS, *B* = .96, *t* (76) = 5.80, *p* = .01. However, the relationship between NCW and SoWB was not significant among those participants who reported high SoWB (+ 1 SD), *B* = .14, *t* (76) = .62, *p* = .54 (See Fig. 4).^1^

## Discussion

The main objective of this research was to analyze how direct (vs. indirect) exposure to a traumatic event affects victims’ health. According to previous research [1], we expected and found that people exposed directly to trauma showed more PTSD symptoms than those exposed in an indirect manner. Directly exposed individuals also generated more negative post-traumatic cognitions about the self and about the world than those exposed indirectly. Both results are consistent with the literature indicating that dysfunctional trauma-related cognitions are strongly related with PTSD symptom severity [43–45]. Finally, as expected, victims exposed directly (vs. indirectly) reported less PWB and SoWB. These results are in line with the *Psycho-Social Model of Trauma* [17, 46], which postulates that traumas caused by intentional violence have accumulating and enduring emotional, social, and political consequences. Therefore, the impact of direct (vs. indirect) traumas on victims’ health was greater, both from a psychopathological and positive function perspective.

Beyond the greater impact of direct (vs. indirect) exposure on victim’s health, as a novel contribution, we expected that well-being would play a different role in the effect of post-traumatic cognitions on victims’ mental health depending on the type of trauma exposure. Regarding indirect exposure, and in line with previous literature [45], we found that this type of exposure generated less dysfunctional trauma-related cognitions (vs. direct exposure). Given the close conceptual relationship expected and detected in our study between negative cognitions about the self and PWB (*r* = −.46, *p* < 0.01), and negative cognitions about the world and social well-being (*r* = −.46, *p* < 0.01), we also expected that well-being would mediate the relationship between post-traumatic dysfunctional cognitions and DTS. The results of our study confirmed this hypothesis, indicating that well-being should be a central element of public policies to protect the general population against indirect exposure to massive trauma (such as terrorist attacks). Moreover, participants who were directly exposed to trauma had different reactions than those indirectly exposed to trauma. According to Emotional Processing Theory [12, 20], a direct exposure to a high-intensity traumatic event should produce strong dysfunctional cognitions. Therefore, in agreement with our predictions, it was expected that well-being moderated the relationship between post-traumatic dysfunctional cognitions and trauma symptoms. Our results confirmed this moderation, indicating that well-being was a “protective factor” for individuals that were directly exposed to trauma. From an applied point of view, these results have interesting implications. First, the important differences found between direct and indirect trauma exposure should be taken into consideration when developing psychological interventions. For example, although the use of classic clinical interventions to prevent the development of PTSD in vulnerable individuals exposed to indirect trauma through the mass media (e.g. based on fear conditioning or extinction models [47]) may be effective, increase the levels of population’s well-being also seems to be an excellent recovery strategy (not only a prevention strategy). According to the literature, three well-being indicators appear to be strongly related to PTSD (or CPTSD), and thus should be the focus of this positive psychological intervention. The first is positive affect. The inability to experience positive emotions (anhedonia; DSM-V PTSD D7 criteria; severe and pervasive problems in affect regulation; ICD-11 CPTSD C1) is present in about two-thirds of PTSD patients, independently of comorbid major depressive disorder [48] (for a review on the possible mechanisms underlying anhedonia in PTSD, see [49]). Therefore, in order to address this issue, interventions such as the expressive writing technique can be applied to focus on positive emotions generated after the trauma (e.g. the support of close social networks such as friends or family, feelings of unity generated in the community). One of the main objectives of this kind of intervention should be to increase positive emotional granularity (i.e., the tendency to represent experiences of positive emotion with precision and specificity) given its crucial role in enhancing coping resources in the face of traumatic events [50]. However, training individuals to employ coping strategies focused on emotion in general (and not only on positive ones), could probably also increase their well-being. According to research on coping strategies [51], individuals who have been indirectly exposed to traumatic events and focus on emotions associated with the stressor, probably cope with trauma much better than individuals who don’t focus on emotions associated with the stressor. One reason for this may be because emotion-focused strategies (vs. problem-focused) are more adaptive in uncontrollable situations like terrorist attacks [52]. It should be noted that, although in many cases clinical psychology and psychiatry focus on disorders and mental health taking as a frame of reference and unit of analysis a subject isolated from its environment [46], there are also different intervention strategies that can be applied not only on an individual or a micro-social level, but also on a macro-social one. The culmination of this proposal is the development of social institutions and positive communities [53].

Finally, regarding direct exposure, our data revealed the important role of negative post-traumatic cognitions about the self and the world in the development and maintenance of PTSD. These results are consistent with previous research indicating that trauma produces negative cognitions about the self and others [30, 54], and that these negative cognitions subsequently increase PTSD in a vicious downward cycle [55], thus reducing well-being over time. Therefore, the development of interventions aimed at modifying these cognitions in individuals directly exposed to traumatic events is critically important. For example, therapies such as prolonged exposure therapy [56], or other forms of cognitive behavioral therapy [57, 58], have been shown to be effective in this area. In the case of trauma caused by terrorist attacks we expect symptoms of persistent difficulties in sustaining relationships and in feeling close to others (ICD-11 CPTSD C3), therefore it would be also interesting to work on negative cognitions that link “others” with the intent of causing harm deliberately. Although work to modify dysfunctional cognitions is essential in post-trauma situations that focus on individuals directly exposed to traumatic events, using positive interventions to increase positive well-being is an excellent prevention strategy. In direct exposure, well-being emerges as a moderator of the relationship between dysfunctional cognitions and psychopathology symptoms, becoming a strong excellent protective factor.

Although the present study made several novel contributions to the literature, some limitations should also be mentioned. The most notable of which is related to our research design. That is, because the topic of this study does not allow the use of an experimental design, this affects our ability to draw causal conclusions regarding the relationships between variables. Another potential limitation is that we have only measured dysfunctional cognitions. Using the approaches of either Epstein [15] or Janoff-Bulmann [13, 16], it would have been interesting to measure the possible rupture of core beliefs caused by trauma exposure (i.e. the world is benign, the world is meaningful, the self is worthy, or people are trustworthy). However, a direct measurement of the process of beliefs’ rupture would have required a longitudinal pre-post trauma design. To obtain a sample with these characteristics is very complex since, as mentioned previously, we cannot manipulate the presence/absence of trauma experimentally. Despite these issues, future research could explore the idea that only direct traumatic events may break the “cognitive homeostasis”, a system that supports the maintenance of positive core beliefs within certain levels of equilibrium.

## Conclusions

The results of our study indicate that the differences found between direct and indirect exposure should be taken into consideration in interventions designed to improve victims’ health. With indirect exposure, social and psychological well-being emerge as a causal factor in the relationship between dysfunctional cognitions and PTSD symptoms. Therefore, positive psychological interventions could be used as prevention and recovery strategies. However, direct exposure to a high-intensity traumatic event causes strong dysfunctional cognitions that in turn constitute an important risk factor in the development of PTSD. With this kind of exposure, social and psychological well-being emerge as protective factors against the development of post-traumatic stress symptoms. Future research could explore the process of core beliefs’ rupture caused by direct and indirect trauma exposure.

## Abbreviations

CI: Confidence Interval
CPTSD: Complex Post-traumatic Stress Disorder
DSM: Diagnostic and Statistical Manual of Mental Disorders
DTS: Davidson Trauma Scale
NCS: Negative Cognitions about Self
NCW: Negative Cognitions about the World
PTCI: Post-traumatic Cognitions Inventory
PTSD: Post-traumatic Stress Disorder
PWB: Psychological Well-being
SB: Self-Blame
SoWB: Social Well-Being

## Footnotes

1 To discard that PWB mediates the relationship between NCS and DTS and SoWB mediates the relationship between NCW and DTS in direct victims, we employed the same procedure as in the previous section. The results of these bootstrapping procedures revealed that the 95% confidence interval of the indirect effect did not include zero (PWB: Indirect Effect a x b = .09, CI95% = from −.03 to .25; SoWB: Indirect Effect a x b = .06, CI95% = from −.07 to .24). Therefore, the mediations by PWB or SoWB are not supported.

## References

[1] Neria Y, Nandi A, Galea S (2008). Post-traumatic stress disorder following disasters: a systematic review. Psychol Med.

[2] Zimering R, Gulliver SB, Knight J, Munroe J, Keane TM (2006). Posttraumatic stress disorder in disaster relief workers following direct and indirect trauma exposure to ground zero. J Trauma Stress.

[3] Schlenger WE, Caddell JM, Ebert L, Jordan BK, Rourke KM, Wilson D (2002). Psychological reactions to terrorist attacks: findings from the National Study of Americans’ reactions to September 11. JAMA.

[4] Silver RC, Holman EA, McIntosh DN, Poulin M, Gil-Rivas V (2002). Nationwide longitudinal study of psychological responses to September 11. JAMA.

[5] Otto MW, Henin A, Hirshfeld-Becker DR, Pollack MH, Biederman J, Rosenbaum JF (2007). Posttraumatic stress disorder symptoms following media exposure to tragic events: impact of 9/11 on children at risk for anxiety disorders. J Anxiety Disord.

[6] Propper RE, Stickgold R, Keeley R, Christman SD (2007). Is television traumatic? Dreams, stress, and media exposure in the aftermath of September 11, 2001. Psychol Sci.

[7] Goodwin R, Lemola S, Ben-Ezra MJ (2017). Media use and insomnia after terror attacks in France. Psychiatry Res.

[8] Suvak M, Maguen S, Litz BT, Silver RC, Holman EA (2008). Indirect exposure to the September 11 terrorist attacks: does symptom structure resemble PTSD?. J Trauma Stress.

[9] American Psychiatric Association (1994). Diagnostic and statistical manual of mental disorders: DSM-IV.

[10] Weathers FW, Keane TM (2007). The criterion a problem revisited: controversies and challenges in defining and measuring psychological trauma. J Trauma Stress.

[11] Ehlers A, Clark DM (2000). A cognitive model of posttraumatic stress disorder. Behav Res Ther.

[12] Foa EB, Kozak MJ (1986). Emotional processing of fear: exposure to corrective information. Psychol Bull.

[13] Janoff-Bulman R (1992). Shattered Asumptions: toward a new psychology of trauma.

[14] Karatzias T, Shevlin M, Fyvie C, Hyland P, Efthymiadou E, Wilson D (2017). Evidence of distinct profiles of posttraumatic stress disorder (PTSD) and complex posttraumatic stress disorder (CPTSD) based on the new ICD-11 trauma questionnaire (ICD-TQ). J Affect Disord.

[15] Epstein S, Ellis A, Grieger R (1991). The self-concept, the traumatic neurosis, and the structure of personality. Handbook of rational emotive therapy.

[16] Janoff-Bulman R (1989). Assumptive worlds and the stress of traumatic events: applications of the schema construct. Soc Cogn.

[17] Blanco A, Blanco R, Díaz D (2016). Social (dis)order and psychosocial trauma: look earlier, look outside, and look beyond the persons. Am Psychol..

[18] Kilpatrick D, Resnick H, Milanak M, Miller M, Keyes K, Friedman M (2013). National estimates of exposure to traumatic events and PTSD prevalence using DSM-IV and DSM-5 criteria. J Trauma Stress.

[19] Morina N, Wicherts JM, Lobbrecht J, Priebe S (2014). Remission from post-traumatic stress disorder in adults: a systematic review and meta-analysis of long term outcome studies. Clin Psychol Rev.

[20] Foa EB, Huppert JD, Cahill SP, Rothbaum BO (2006). Emotional processing theory: an update. Pathological anxiety: emotional processing in etiology and treatment.

[21] Foa E, Ehlers A, Clark D, Tolin D, Orsillo S (1999). The post-traumatic cognition inventory (PTCI): development and validation. Psychol Assess.

[22] Ryff C (1989). Happiness is everything, or is it? Explorations on the meaning of psychological well-being. J Pers Soc Psychol.

[23] Keyes C (1998). Social Well-Being. Soc Psychol Q.

[24] Keyes C (2005). Mental illness and/or mental health? Investigating axioms of the complete state model of health. J Consult Clin Psychol.

[25] Díaz D, Stavraki M, Blanco A, Bajo M. 11-M victims 3 years after Madrid terrorist attacks: looking for health beyond trauma. J Happiness Stud. 2017; 10.1007/s10902-016-9842-x

[26] Dunmore, E, Ehlers A, Clark DM. A prospective investigation of the role of cognitive factors in persistent Posttraumatic Stress Disorder (PTSD) after physical or sexual assault. Behav Res Ther. 2001;39:1063–84. 10.1016/s0005-7967(00)00088-7 11520012

[27] Rosenthal R (2002). Covert communication in classrooms, clinics, courtrooms, and cubicles. Am Psychol.

[28] Davidson JRT, Book SW, Colket JT, Tupler LA, Roth S, David D (1997). Assessment of a new self-rating scale for post-traumatic stress disorder. Psychol Med.

[29] Bobes J, Calcedo-Barba A, García M, Francois M, Rico-Villademoros F, González M (2000). Evaluation of the psychometric properties of the Spanish version of five questionnaires for the assessment of post-traumatic stress disorders. Actas Esp Psiquiatr.

[30] Blanco A, Díaz D, Gaborit M, Amaris MC (2010). World Schema and self Schema: the posttraumatic cognitions inventory (PTCI) in Hispanic population. Rev Lat Am Psicol.

[31] Díaz D, Rodríguez-Carvajal R, Blanco A, Moreno-Jiménez B, Gallardo I, Valle C (2006). Spanish adaptation of the psychological well-being scales (PWBS). Psicothema.

[32] Díaz D, Blanco A, Durán MM (2011). The structure of well-being: the empirical encounter of three traditions. Rev Psicol Soc.

[33] Van Dierendonck D, Díaz D, Rodríguez-Carvajal R, Blanco A, Moreno-Jiménez B (2008). Ryff’s six-factor model of psychological well-being, a Spanish exploration. Soc Indic Res.

[34] Blanco A, Díaz D (2005). Social well-being: theoretical structure and measurement. Psicothema..

[35] Díaz D, Blanco A, Bajo M, Stavraki M (2015). Fatalism and well-being across hispanic cultures: the social fatalism scales (SFS). Soc Indic Res.

[36] Baron RM, Kenny DA (1986). The moderator-mediator variable distinction in social psychological research: conceptual, strategic and statistical considerations. J Pers Soc Psychol.

[37] Hayes AF (2013). Introduction to mediation, moderation, and conditional process analysis.

[38] Preacher KJ, Hayes AF (2004). SPSS and SAS procedures for estimating indirect effects in simple mediation models. Behav Res Methods Instrum Comput.

[39] Shrout PE, Bolger N (2002). Mediation in experimental and nonexperimental studies: new procedures and recommendations. Psychol Methods.

[40] Foa EB, Rothbaum BO (1998). Treating the trauma of rape: cognitive-behavioral therapy for PTSD.

[41] McCann IL, Pearlman LA (1990). Vicarious traumatization: a framework for understanding the psychological effects of working with victims. J Trauma Stress.

[42] Resick PA, Schnicke MK (1992). Cognitive processing therapy for sexual assault victims. J Consult Clin Psychol.

[43] Dunmore E, Clark DM, Ehlers A (2001). A prospective investigation of the role of cognitive factors in persistent posttraumatic stress disorder (PTSD) after physical or sexual assault. Behav Res Ther.

[44] McLean CP, Yeh R, Rosenfield D, Foa EB (2015). Changes in negative cognitions mediate PTSD symptom reductions during client-centered therapy and prolonged exposure for adolescents. Behav Res Ther.

[45] Moser JS, Hajcak G, Simons RF, Foa EB (2007). Posttraumatic stress disorder symptoms in trauma-exposed college students: the role of trauma related cognitions, gender and negative affect. J Anxiety Disord.

[46] Blanco A, Díaz D. The twofold face of fatalism: collectivist fatalism and individualist fatalism. Psicothema. 2007;19:552–8. 17959106

[47] Neria Y, Sullivan GM (2011). Understanding the mental health effects of indirect exposure to mass trauma through the media. JAMA.

[48] Carmassi C, Akiskal HS, Bessonov D, Massimetti G, Calderani E, Stratta P (2014). Gender differences in DSM-5 versus DSM-IV-TR PTSD prevalence and criteria comparison among 512 survivors to the L’Aquila earthquake. J Affect Disord.

[49] Nawijn L, van Zuiden M, Frijling JL, Koch SB, Veltman DJ, Olff M (2015). Reward functioning in PTSD: a systematic review exploring the mechanisms underlying anhedonia. Neurosci Biobehav Rev.

[50] Tugade MM, Fredrickson BL, Barrett LF (2004). Psychological resilience and emotional granularity: examining the benefits of positive emotions on coping and health. J Pers.

[51] Littleton H, Horsley S, John S, Nelson DV (2007). Trauma coping strategies and psychological distress: a meta-analysis. J Trauma Stress.

[52] Folkman S, Moskowitz JT (2004). Coping: pitfalls and promise. Annu Rev Psychol.

[53] Seligman M, Csikszentmihalyi M (2000). Positive psychology: an introduction. Am Psychol.

[54] Foa EB, Dancu CV, Hembree EA, Jaycox LH, Meadows EA, Street GP (1999). A comparison of exposure therapy, stress inoculation training, and their combination for reducing posttraumatic stress disorder in female assault victims. J Consult Clin Psychol.

[55] Shahar G, Noyman G, Schnidel-Allon I, Gilboa-Schechtman E (2013). Do PTSD symptoms and trauma-related cognitions about the self constitute a vicious cycle? Evidence for both cognitive vulnerability and scarring models. Psychiatry Res.

[56] Powers MB, Halpern JM, Ferenschak MP, Gillihan SJ, Foa EB (2004). A meta-analytic review of prolonged exposure for posttraumatic stress disorder. Clin Psychol Rev.

[57] Klein B, Mitchell J, Abbott J, Shandley K, Austin D, Gilson K (2010). A therapist-assisted cognitive behavior therapy internet intervention for posttraumatic stress disorder: pre-, post- and 3-month follow-up results from an open trial. J Anxiety Disord.

[58] Cruz-Feliciano MA, Miranda-Diaz C, Fernandez-Santos DM, Orobitg-Brenes D, Hunter-Mellado RF, Carrion-Gonzalez IS (2017). Quality of life improvement in latinas receiving combined substance use disorders and trauma-specific treatment: a cohort evaluation report. Health Qual Life Outcomes.

